# Physiological and Molecular Aspects of Two *Thymus* Species Differently Sensitive to Drought Stress

**DOI:** 10.3390/biotech11020008

**Published:** 2022-03-23

**Authors:** Mohsen Ashrafi, Mohammad-Reza Azimi-Moqadam, Ehsan MohseniFard, Farid Shekari, Hossein Jafary, Parviz Moradi, Mariachiara Pucci, Giulia Abate, Andrea Mastinu

**Affiliations:** 1 Department of Agronomy and Plant Breeding, Faculty of Agriculture, University of Zanjan, Zanjan 45195-313, Iran; m.ashrafi@znu.ac.ir (M.A.); mohsenifard.ehsan@znu.ac.ir (E.M.); shekari@znu.ac.ir (F.S.); 2 Research Division of Plant Protection, Zanjan Agricultural and Natural Resources Research and Education Centre, AREEO, Zanjan 45195-313, Iran; h.jafary@areeo.ac.ir; 3 Research Division of Natural Resources, Zanjan Agricultural and Natural Resources Research and Education Centre, AREEO, Zanjan 45195-313, Iran; 4 Department of Molecular and Translational Medicine, University of Brescia, 25123 Brescia, Italy; m.pucci003@unibs.it (M.P.); andrea.mastinu@unibs.it (A.M.)

**Keywords:** *Thymus*, water stress, histone deacetylase-6, acetyl CoA synthetase, succinyl CoA ligase, pyruvate decarboxylase-1, qRT-PCR

## Abstract

Drought is one of the most important threats to plants and agriculture. Here, the effects of four drought levels (90%, 55%, 40%, and 25% field capacity) on the relative water content (RWC), chlorophyll and carotenoids levels, and mRNA gene expression of metabolic enzymes in *Thymus vulgaris* (as sensitive to drought) and *Thymus kotschyanus* (as a drought-tolerant species) were evaluated. The physiological results showed that the treatment predominantly affected the RWC, chlorophyll, and carotenoids content. The gene expression analysis demonstrated that moderate and severe drought stress had greater effects on the expression of histone deacetylase-6 (HDA-6) and acetyl-CoA synthetase in both *Thymus* species. Pyruvate decarboxylase-1 (PDC-1) was upregulated in *Thymus vulgaris* at high drought levels. Finally, succinyl CoA ligase was not affected by drought stress in either species. Data confirmed water stress is able to alter the gene expression of specific enzymes. Furthermore, our results suggest that PDC-1 expression is independent from HDA-6 and the increased expression of ACS can be due to the activation of new pathways involved in carbohydrate production.

## 1. Introduction

Drought is an abiotic environmental stressor that can limit plant growth, yield, and productivity [1,2]. During drought, relative water content (RWC) is one parameter that best describes the water status of plants [3,4,5]. RWC is a factor that assesses both the effect of the soil−plant−atmosphere water continuum and the effect of membrane osmotic potentials. Indeed, different cultivars (but plants of the same species) with the same foliar water potential can have different RWCs [6]. RWC decreases proportionally with the decrease in water availability [7]. Species sensitive to drought showed lower RWC values than drought-resistant species.

Water stress can alter not only the plant osmotic potential, but also the stomata opening, which in turn affects the photosynthetic efficiency [8].

The decrease in photosynthetic yield in plants subjected to water stress is due to the reduction of the chlorophyll content [8]. The persistence of drought determines the closure of the stomata in order to avoid water loss. This event blocks the entry of CO_2_ necessary for photosynthesis, with a consequent loss of chlorophyll. The leaves turn yellow because of the dominance of carotenoids compared to the green pigments of chlorophyll [9].

For aromatic and medicinal plants, a strong water stress occurring before the flowering phase (vegetative period) can generate lower plants with smaller leaf areas, as observed in the genus *Mentha*, *Achillea*, *Calendula,* and *Melissa* [10,11,12,13]. The reduction of the foliar apparatus leads to a reduced production of organic matter because of the lower photosynthetic yield [14]. However, some plants have developed resistance and acclimatization mechanisms to drought, such as some *Thymus* species [15].

*Thymus* is one of the most important genera of the Lamiaceae family for the number of species it contains [16]. It has been used throughout history as a medicinal, aromatic, and spicy plant [17]. Thyme is distributed in different areas worldwide, such as Mediterranean regions with scarcely rainy climates [18]. Some *Thymus* species have developed different resistance-levels to water stress. In particular, previous works have shown that *Thymus kotschyanus* (*T. kotschyanus*) under severe drought stress conditions could grow and survive more than *Thymus vulgaris* (*T. vulgaris*) [19,20]. The different drought tolerability of *Thymus* species could be derived from the different regulation of primary and secondary metabolism. In a previous experiment, we performed a ^1^H-NMR metabolomics analysis on *T. kotschyanus* and *T. vulgaris,* and found that succinic acid and acetic acid concentrations were differently affected by drought in both species [19]. Studies have revealed that in water stress conditions, succinic acid and acetic acid concentrations are involved in growth and survivability, respectively [21,22]. In particular, succinic acid in plants acts as an osmotic regulator, favoring a greater adaptability to conditions of abiotic stress, such as drought [23]. At the same time, severe drought affects succinic acid levels in plants [23]. Acetic acid improves the plant’s resistance to drought by acting on the hormonal response regulated by jasmonates and abscisic acid [24,25]. Our previous study revealed that *T. kotschyanus* has higher levels of succinic acid and lower levels of acetic acid than *T. vulgaris* [19]. This could explain the different responses to water stress of the two *Thymus* species. To date, little is known about the gene regulation of succinic acid and acetic acid synthesis enzymes in *Thymus* under water stress conditions. Environmental stimuli (light, water, mineral salts, and parasites) can influence the regulation of gene expression and also affect chromatin and histone proteins [26]. In plants, mutations on the epigenetic regulator histone deacetylase-6 (HDA-6) appear to improve survival in drought conditions [27]. This response is associated with the expression of genes involved in acetic acid biosynthesis. Therefore, in conditions of water stress, there would be a relationship between HAD-6 and regulation of genes involved in acetic acid synthesis [28].

The main aim of this manuscript was initially to evaluate some physiological parameters of two species of *Thymus* differently resistant to drought. Subsequently, the gene expression of some markers directly or indirectly involved in the metabolism of succinic acid and acetic acid was also evaluated. In particular, the gene expression of succinyl CoA ligase (SCL), pyruvate decarboxylase-1 (PDC-1), acetyl-CoA synthetase (ACS), and histone deacetylase-6 (HDAC-6) in *T. kotschyanus* and *T. vulgaris* subjected to different degrees of drought was investigated.

## 2. Materials and Methods

### 2.1. Plant Material, Growth Condition, and Treatment

*Thymus vulgaris* and *Thymus kotschyanus* seeds were purchased from Pakan Bazr-e-Esfahan Company (Esfahan, Iran).

The seeds were washed and sanitized following a previously reported protocol [8,29]. Subsequently, the seeds were sown in 10 cm diameter pots filled with approximately 285 g of soil mixture (ratio of 0.5:1:1:2 of perlite/sand/vermicompost/compost, respectively). The experiment was performed in triplicate per treatment and was conducted in a greenhouse with a day/night period of 18/6 h and an average day/night temperature of 24/20 °C. Initially, the pots were irrigated daily at 90% of the field capacity (FC) for two months. Subsequently, four levels of irrigation regimen were applied, including normal irrigation (90% FC) and varying degrees of stress: mild (55% FC), moderate (40% FC) and severe (25% FC). Two days after reaching severe stress, the leaves were harvested for further analysis.

### 2.2. Physiological Measurements 

Relative water content (RWC) was measured by the method of Barrs and Weatherley [30,31]. Briefly, the thyme leaflets were collected and weighed (fresh weight (FW)) and then soaked overnight in distilled water at 4 °C. After cold incubation, the leaves were dried with paper and weighed (turgid weight (TW)) and subsequently dried in an oven at 80 °C for 48 h. The dry weight (DW) of the leaves was then measured. The relative water content of the leaves was calculated using Equation (1).

RWC = (FW − DW)/(TW − DW)) × 100 (1)

The chlorophyll and carotenoids contents were measured using the Arnon and Lichtenthaler and Wellburn methods, respectively [29,30,32]. In brief, about 100 mg of leave samples were ground in liquid nitrogen using a mortar and pestle. Then, exactly 4 mL of 80% acetone was added to the ground sample and the resulting solution was centrifuged at 6000 rpm for 10 min at 4 °C. The aqueous phase was placed in a glass tube. In the next step, the absorbance at 663 nm (for chlorophyll a), 645 nm (for chlorophyll b), and at 470 nm (for carotenoids) were determined spectrophotometrically.

Finally, the following equations

Chlorophyll a = (19.3 × A663−0.86 × A645) V/100 W(2)

Chlorophyll b = (19.3 × A645−3.6 × A663) V/100 W(3)

Carotenoids = 100(A470) − 3.27(mg chl. a) − 104(mg chl. b)/227(4)

were used for calculation of chlorophylls a and b, as well as carotenoids, in which V= aqueous phase volume; A= absorbance at 663, 645, and 470 nm; and W= fresh weight of sample.

### 2.3. Total RNA Isolation, Quality Controls, and First Strand cDNA Synthesis

The total RNA was isolated from the leaves (100 mg) of *T. kotschyanus* and *T. vulgaris* using the method of Chomczynski and Sacchi [33]. The RNA quality and concentration were determined using a Nanodrop ND-2000 spectrophotometer (Thermo Scientific, Waltham, MA, USA). Subsequently, 4.0 µg of total RNA was treated with DNase I (Thermo Fisher Scientific, Waltham, MA, USA) and was reverse-transcribed using Hyperscript RT-PCR Mastermix (GeneAll, Seoul, South Korea) and the oligo-dT primer according to the instructions of the manufacturer. Finally, the cDNA was diluted to a final concentration of 100 ng/µL with sterile MilliQ water prior to te qRT-PCR analysis.

### 2.4. Gene Selection, Amplification, and Direct Sequencing

A set of four genes were selected, including succinyl CoA ligase (SCL), pyruvate decarboxylase-1 (PDC-1), acetyl-CoA synthetase (ACS), and histone deacetylase-6 (HDA-6).

The mRNA sequence of *SCL, ACS, PCD-1,* and *HDA-6* genes was not available for these two species; therefore, the conserved domain of known sequences of the *Lamiids* clade was used for designing of degenerate primers (Table 1).

PCR was used for amplification of the selected segment of the mentioned genes. PCR amplification reactions (25 μL) contained 2.5 μL of 10x enzyme buffer, 1 mM MgCl_2_, 200 μM each of dATP, dCTP, dGTP, and dTTP (Sinaclon, Tehran, Iran), 0.4 μM each primer (Macrogen, Seoul, South Korea) (Table 1), 1 unit of Taq polymerase (Sinaclon, Tehran, Iran), and 100 ng of cDNA. The PCR cycles consisted of initial denaturation at 94 °C for 4 min, followed by 35 cycles of denaturation at 94 °C for 60 s, annealing at 46–56 °C for 45 s (Table 1), and extension at 72 °C for 1 min with a final extension at 72 °C for 5 min. The amplicons were later resolved on 1.2% agarose gel and then reverse primers were used for direct sequencing using the Sanger method (Macrogen, Seoul, South Korea). Next, these sequences were deposited on the NCBI database (*SCL*: MH444601, MH444602; *ACS*: MH444599, MH444600; *PDC-1*: MH602964, MH602965; and *HDA-6*: MH444603, MH570448).

### 2.5. qRT-PCR

The qRT-PCR primers (Table 2) for SCL, PDC, ACS, and HDA-6 were designed with premier 5 software (Premier Biosoft, Palo Alto, CA, USA). The reaction mix (20 µL) contained 100 ng of cDNA, 0.2 µM of each primer, and 4 µL of HOT FIREPol^®^ EvaGreen^®^ qPCR Mix Plus (no ROX) (Solis BioDyne, Tartu, Estonia). The reaction was performed on the Rotor-Gene Q Real-Time PCR system (Qiagen, Hilden, Germany). The negative control had no cDNA, which did not produce any noticeable fluorescence signals from the reaction. The qRT-PCR conditions were set as follows: initial denaturation for 30 s at 95 °C, followed by 40 cycles of denaturation at 95 °C for 15 s, annealing at 55 °C for 20 s, and extension at 72 °C for 15 s. After the amplification cycles, the melting curves for each reaction were evaluated to confirm the specificity of the amplified products. EF-1A and GAPDH for *T. kotschyanus* and Act and GAPDH for *T. vulgaris* were used as housekeeping genes. Relative quantification was performed using the comparative Ct method.

### 2.6. Experimental Design and Statistical Analysis

The experimental design was a factorial experiment in completely randomized design, in which water treatment and plant species were the factors. Analyses of variance (ANOVA) were computed for physiological criteria. ANOVA and mean comparison were performed using R and Agricolaa Package.

## 3. Results

### 3.1. Physiological Measurements

**Figure 1:**
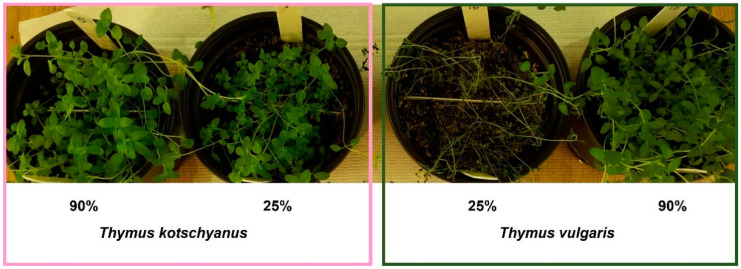
Crops of *Thymus kotschyanus* (**left**) and *Thymus vulgaris* (**right**) in two different irrigation conditions: normal irrigation (90%) and severe water stress (25%). These two examples show the greater sensitivity of *T. vulgaris* to water stress compared to *T. kotschyanus*.

The ANOVA analysis highlighted significant differences between the two *Thymus* species for all of the physiological parameters analyzed (Table 3). This analysis allowed for evaluating the effects of the four irrigation regimes (degrees freedom: 3) on the two species of *Thymus* (degrees freedom: 1). In particular, the effects of the different irrigation regimes led to a significantly different relative water content (RWC) in both *T. vulgaris* and *T. kotschyanus*. This significant response between the two *Thymus* species was also observed in the total chlorophyll, carotenoids, and chlorophyll/carotenoids ratio. Figure 1 shows the different response to severe water stress for the two *Thymus* species. In particular, the following two extreme irrigation conditions are shown: 25% FC greater water stress and 90% FC optimal irrigation.

**Figure 2:**
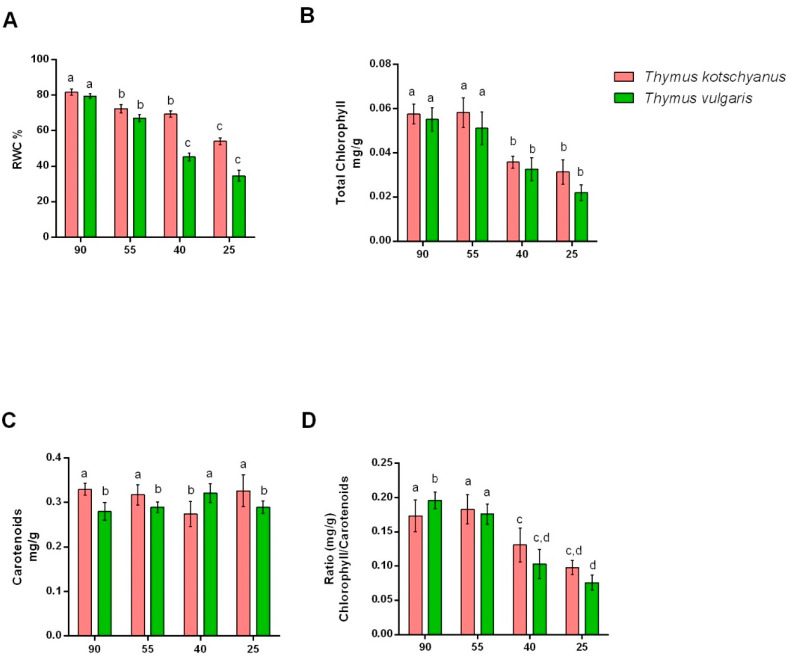
Effect of different irrigation regimes, normal irrigation (90%), mild water stress (55%), moderate water stress (40%), and severe water stress (25%) on RWC (**A**), total chlorophyll (**B**), carotenoids content (**C**), and chlorophyll/carotenoids ratio (**D**) in *Thymus kotschyanus* and *Thymus vulgaris*. Data are represented as mean ± S.E.M (bar plot). The bars with the same letter are not significantly different according to the corrected Benjamini−Hochberg *t*-test *p* ≤ 0.05.

From the observation of the different irrigation regimes, it was observed that reducing irrigation lowers RWC in both species of thyme (Figure 2A). In particular, *T. vulgaris* under moderate water stress conditions showed a significant reduction compared to both normally irrigated plants and to *T. kotschyanus* (Figure 2A). As for chlorophyll, a significant loss of 40% of irrigation was observed in both species of *Thymus* (Figure 2B). Different irrigation regimes did not considerably affect the carotenoids content in the two species of *Thymus* (Figure 2C). However, data suggest the two species have different amounts of carotenoids under normal irrigation conditions (Figure 2C). Finally, the chlorophyll/carotenoids ratio decreased significantly with the increase in water stress, especially in *T. vulgaris* (Figure 2D). These data confirm that *T. vulgaris* is more sensitive to drought than *T. kotschyanus.*

### 3.2. Gene Expression Analysis

**Figure 3:**
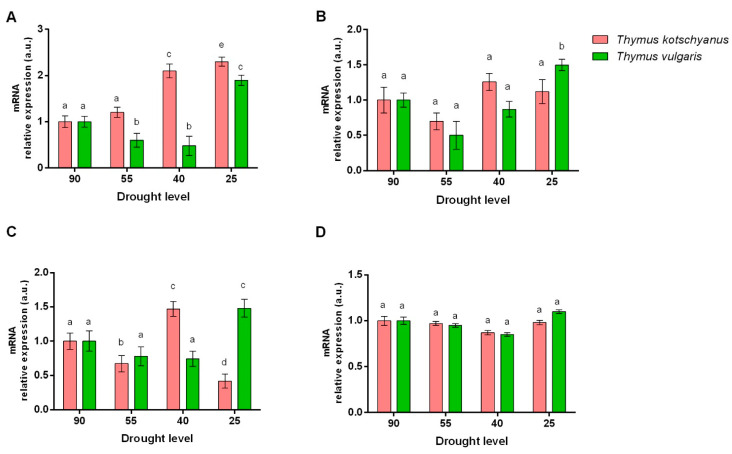
Effect of drought stress on the relative expression of histone deacetylase-6 (HDA-6) (**A**), pyruvate decarboxylase-1 (PDC-1) (**B**), acetyl CoA synthetase (ACS) (**C**), and succinyl CoA ligase (SCL) (**D**). Data are represented as mean ± S.E.M (bar plot). The bars with the same letter are not significantly different according to the corrected Benjamini−Hochberg *t*-test *p* ≤ 0.05.

In *T. kotschyanus,* mild drought stress had no effects on the expression of *HDA-6* gene, but moderate and severe drought stress up-regulated its expression. Unlike *T. kotschyanus*, in *T. vulgaris,* mild and moderate drought stress down-regulated the expression of *HDA-6,* and only severe drought stress-induced its expression (Figure 3A). No significant differences were found in PDC-1 mRNA expression in *T. kotschyanus* at different degrees of water stress. On the other hand, *T. vulgaris* showed a significant increase in PDC-1 only under severe drought stress conditions (25%) compared to other irrigation levels (Figure 3B). In *T. kotschyanus*, each degree of drought significantly modified the ACS gene expression. In detail, mild and severe drought stress reduced the expression of ACS mRNA, while moderate drought stress increased its levels. Unlike *T. kotschyanus*, in *T. vulgaris,* only severe drought stress affected the ACS mRNA expression by increasing it (Figure 3C). The expression of SCL was not affected by drought stress in any of the species (Figure 3D).

## 4. Discussion

Climate change has significantly altered rainfall throughout the years by increasing drought in ecologically fragile areas of the Earth. Drought is the abiotic stress that most affects plant growth [34]. The tolerance and sensitivity of plants to drought depends on (i) intrinsic factors such as the species, cultivars, and growth stage of the plant, and (ii) extrinsic factors such as the duration and intensity of drought stress [35,36]. In this manuscript, two species of *Thymus*, *T. vulgaris* and *T. kotschyanus*, were subjected to different degrees of water stress, and some physiological and molecular parameters were measured. The *Thymus* genus includes species with different sensitivities to drought [37]. In particular, it has been previously observed that *Thymus carmanicus* is sensitive to drought; *T. vulgaris* is semi-sensitive to drought; whereas *T. kotschyanus*, *Thymus daenensis,* and *T. vulgaris* (cultivar Spain) are semi-tolerant to drought [19,38].

Here, it has been confirmed that *T. vulgaris* is more sensitive to water stress than *T. kotschyanus*. Indeed, the initial relative water content (RWC) is significantly lower in *T. vulgaris* than in *T. kotschyanus*. This is confirmed by the ability of drought-resistant plants to develop mechanical defenses (isolated) to conserve water in their own tissues [39,40]. Indeed, a greater thickening of the leaf epidermis and a lower stomatal distribution are the best strategies used by plants in arid climates [41]. *T. kotschyanus* has a multilayered epidermis that allows for a lower waste of water when compared to *T. vulgaris* [16]. Both species have developed strategies to counteract water loss or drought, but *T. vulgaris* is more vulnerable. As expected, the four irrigation regimes resulted in a reduction in RWC because of the decreased water availability. RWC reduction causes an alteration in the transpiration processes, leading to stomata closure [42]. This event determines a reduction in the photosynthetic processes due to a decrease in CO_2_ [43]. In this regard, our data highlighted that both *Thymus* species exhibited a reduced photosynthetic performance due to water stress, confirming what has been observed by other authors [15,44,45].

Furthermore, with the increase in water stress, a sharp drop in the total chlorophyll content of both *Thymus* species was observed. The decrease in chlorophyll under drought stress was mainly the result of damage to chloroplasts caused by the increase in reactive oxygen species (ROS) [31,46,47]. The reduction was more evident starting from a moderate water stress level (40%). In fact, in the initial stages of mild drought, ROS triggered molecular signaling regulated by some hormones (such as abscisic acid) that protect against cell damage [7]. This protective effect is reduced with increasing drought.

Drought levels did not change the carotenoids content of both *Thymus* species. Carotenoids play an important role in counteracting oxidative stress and promoting drought resistance [48,49]. For this reason, the significant differences observed already at the basal conditions (normal irrigation, 90%) may explain the different responses to water stress of the two Thymus species. Indeed, the high levels of carotenoids observed in *T. kotschyanus* may explain its better resistance to severe drought regimes.

Regarding the molecular data, the severe drought level induced HDA-6 expression in both *Thymus* species. HDA-6 levels were statistically higher in *T. kotschyanus* than in *T. vulgaris*. Previous work revealed that *HDA* genes play important roles in regulating the plant gene expression [50]. *HDA-6* exerts various roles, including the suppression of specific genes [22], by removing lysine from histones H3 and H4. This enzyme binds to target genes via transcription factors that bind to DNA in large multiprotein transcriptional complexes [50]. Kim and colleagues indicated that *HDA-6* mutant plants are more tolerant to drought than wild-type plants due to their inability to suppress the expression of acetaldehyde dehydrogenase and *PDC-1* genes, and consequently produce mostly acetic acid [22]. The expression of *PDC-1* was also investigated in the present study. The results revealed that the expression of *PDC-1* was not affected by drought stress in *T. kotschyanus,* whereas in *T. vulgaris,* severe drought stress significantly increased the expression of this gene. The increase in PDC-1 expression is related to the fermentation process and to anaerobic and aerobic phenomena. The increase in PDC-1 in *T. vulgaris* could be related to the absence of oxygen due to the reduced photosynthetic activity resulting from substantial stomatal closure causing less CO_2_. As HDA-6 is increased in *T. kotschyanus* (but not PDC-1), the upregulation of the two genes may not always be associated, as observed in *T. vulgaris*.

The results indicate that water stress increased ACS expression in both species, as reported by Agrawal and colleagues [51]. It is also known that acetic acid can be converted to acetyl-CoA by acetyl-CoA synthetase. The acetyl-CoA produced could be used in the Krebs cycle, both as an energy producer and as an electron carrier [47,51]. Furthermore, ACS are fundamental enzymes for producing carbohydrates from lipids (glyoxylate cycle) [52]. During the glyoxylate cycle, acetyl-CoA can release succinate, which can be transformed into carbohydrates through other metabolic pathways [52]. Usually, the glyoxalate cycle is activated when photosynthesis is not active (seeds). The loss of photosynthetic pigments could trigger this alternative metabolic pathway to produce carbohydrates. In our data, the increased expression of the ACS enzyme occurs earlier in *T. kotschyanus* (level 40) than in *T. vulgaris* (level 25). It could be hypothesized that this mechanism underlies a better tolerability to water stress in *T. kotschyanus*.

Our previous H^1^-NMR metabolomics study [19] showed that drought stress significantly affects the succinic acid concentration, so we investigated the SCL gene expression. The SCL gene encodes the succinyl-CoA ligase enzyme, which converts succinyl-CoA into succinic acid [53]. Unlike the results of Agrawal and colleagues, who observed an increase in succinyl-CoA ligase in drought conditions [51], our results indicated that, in both species, the expression of SCL is not affected by water stress. Thus, the observed differences in succinic acid concentration levels may be due to changes in the expression and/or activity of succinate dehydrogenase or isocitrate lyase.

## 5. Conclusions

The response of plants to water stress is regulated by both genetic and environmental factors. In this manuscript, two species of *Thymus* with different sensitivities to water stress have been selected. Physiological alterations in terms of water potential and photosynthesis have been confirmed. In addition, the gene expression of markers involved in the molecular pathways of the drought response have been evaluated. Our data showed that water stress increases the expression of HDA-6 in both *Thymus* species. This aspect will need to be further explored given the countless genes regulated by HAD-6. In addition, the lack of water causes macroscopic changes (such as the closure of stomata) that can affect the main metabolic pathways. Notably, an increased expression of the PDC-1 enzyme was observed mainly in *T. vulgaris*, and ACS was expressed early in *T. kotschyanus*. These data are the starting point for evaluating the molecular responses activated by different *Thymus* species to water stress in the future. Additional genes will need to be included in the future in order to better define the drought response of plants.

## Figures and Tables

**Table 1:** Characteristics of degenerate primers and GenBank accession ID of sequences used for finding the conserved region.

Gene	Primer Sequence (5′ → 3′)		Accession Id	Product Length	Ta *
*SCL*	Forward Reverse	TCTATGTHCCTCCDCCWTTTGC TCTGCYGTRCCACCRATYTCAC	NM_001324746.1 XM_006350317.2 XM_015211494.1 NM_001247645.2 XM_019302845.1	436	53
*PDC*	Forward Reverse	CAAACTGTBACTTGCTAYCAGG GCCCRTCRTGGATYTCTACTTC	JF775376.1 XM_023031688.1 XM_006362311.2 XM_019399015.1	1060	52
*ACS*	Forward Reverse	MABATAGAGTGGTTCAARGGTG DAARGTAGCAGAGCCAGGTTTC	XM_012988613.1 XM_020698950.1 KJ531400.1 XM_022985897.1 XM_019315775.1	1143	52
*HDA-6*	Forward Reverse	ATCGGCGAYTACTACTACGG ATGACYTTYTGGATKATGGGAC	XM_023030385.1 XM_011102990.2 XR_002286330.1 XM_020691311.1 XM_012973837.1	698	52

* Annealing temperature.

**Table 2:** Primer sequences for genes used in RT-qPCR and their features.

Gene	Primer Sequence (5′ → 3′)		Product Length nt	Efficiency	TA *
*SCL*	Forward	CTGGTTTGTGAATGTATCCC	105	1.9	55
	Reverse	TGAATCAGCAGAAAAAGACTC			
*PDC*	Forward	CCGATGAAATGAGGGTGA	123	2.03	55
	Reverse	GAAACTGTGTGTGGCGAAAG			
*ACS*	Forward	TCAAGCAAACATCTACGACTG	99	2.06	55
	Reverse	CTGTAAAACGAGGACCCAAG			
*HDA-6*	Forward	GTTTCAATGTTGGAGAGGACTG	165	2	55
	Reverse	AGGACTCGCTCTTCTTCGC			
Act	Forward	AGCAACTGGGATGATATGGAG	111	1.92	55
	Reverse	CTTGGGGTTAAGAGGAGCC			
GAPDH	Forward	AACGGAAAGTTGACTGGTATG	126	1.98	55
	Reverse	TGACTCCTCCTTGATGGCA			
EF-1A	Forward	AGATCGGAAATGGTTATGCTC	94	1.95	55
	Reverse	GACCTCCTGTCAATCTTCGT			

* Annealing temperature.

**Table 3:** ANOVA of physiological criterion.

Source of Variation	Degrees Freedom	Mean Squares			
		RWC	Total Chlorophyll	Carotenoids	Chlorophyll/Carotenoids
Species (*T. vulgaris* and *T. kotschyanus*)	1	235.61 **	0.004972 **	0.019777 **	0.025516 **
Treatment (four irrigation regimes)	3	1088.79 **	0.001462 **	0.002133 ^ns^	0.012725 **
Species × Treatment	3	2.15 ^ns^	0.000382 ^ns^	0.004442 ^ns^	0.001099 ^ns^
Residuals	24	14.82	0.000147	0.001748	0.001015

** and ^ns^ are significant at *p* ≤ 0.01 and not significant, respectively.

## Data Availability

The data presented in this study are available upon request from the corresponding author.
